# Physiological shear stress suppresses apoptosis in human pulmonary microvascular endothelial cells

**DOI:** 10.14814/phy2.70269

**Published:** 2025-03-20

**Authors:** Simin Yan, Nicolas M. Philip, Samuel T. Murray, Xin Yun, Michael P. Croglio, Karthik Suresh, Mahendra Damarla, Larissa A. Shimoda, Todd M. Kolb

**Affiliations:** ^1^ Division of Pulmonary and Critical Care Medicine Johns Hopkins University School of Medicine Baltimore Maryland USA

**Keywords:** apoptosis, human pulmonary microvascular endothelial cells, phosphatidylinositol 3‐kinase, shear stress

## Abstract

Physiological shear stress contributes to maintaining endothelial cell homeostasis, including suppression of apoptosis. In the pulmonary circulation, diseases such as pulmonary embolism and pulmonary hypertension result in alterations in shear stress. Shear stress has been reported to suppress endothelial apoptosis through phosphatidylinositol 3‐kinase (PI3K) activation, but evidence from human pulmonary microvascular endothelial cells (PMVECs) is lacking. We hypothesized that physiological shear stress activates PI3K to reduce apoptosis in human PMVECs. We utilized the orbital shaker model of shear stress to test our hypothesis. Apoptosis was evaluated by measuring chromatin condensation, caspase 3/7 activity, and DNA fragmentation. We found that shear stress caused a rapid and sustained increase in protein kinase B (Akt) phosphorylation, a surrogate for activated PI3K, in human PMVECs. Under static conditions, PI3K inhibition with LY294002 or challenge with the kinase inhibitor staurosporine (STS) induced apoptosis in PMVECs. Following exposure to shear stress for 24 h, LY294002‐ and STS‐induced apoptosis was reduced. The anti‐apoptotic effect of shear stress in STS‐challenged cells was reversed by PI3K inhibition. We conclude that physiological shear stress increases PI3K/Akt activity and suppresses apoptosis in normal human PMVECs.

## 1 INTRODUCTION

Hemodynamic shear stress describes the flow‐associated frictional force that acts directly on the apical surface of the endothelium in blood vessels. Depending on the magnitude, pattern, and spatiotemporal distribution of this force, shear stress can regulate a variety of endothelial cell (EC) behaviors, including apoptosis (Davies, 2009; Li et al., 2005). In the pulmonary circulation, shear stress may be an important regulator of endothelial homeostasis. Alterations in pulmonary blood flow (and associated shear stress) may be important contributors to pulmonary vascular diseases, including pulmonary embolism (PE), pulmonary arterial hypertension (PAH; Barker et al., 2015; Odagiri et al., 2016; Schäfer et al., 2017, 2019; Tang et al., 2012), and chronic thromboembolic pulmonary hypertension (CTEPH; Tsubata et al., 2023).

The pulmonary endothelium is composed of heterogeneous cell populations, broadly known as macro‐ and microvascular ECs (Stevens, 2005, 2011). Microvascular ECs exhibit different apoptotic susceptibility from macrovascular ECs (Lu, 2008; Lu et al., 2009) and importantly represent target cells in diseases like PAH (Stevens, 2005). In other endothelial cell types (i.e., human umbilical vein endothelial cells; HUVECs), exposure to shear stress with a magnitude in the physiological range suppresses apoptosis (Dimmeler et al., 1996; Walshe et al., 2013), in part due to activation of the phosphatidylinositol 3‐kinase (PI3K)/ protein kinase B (Akt) signaling pathway (Dimmeler et al., 1998; Kim & Woo, 2018). Whether and how physiological shear stress regulates apoptosis in human pulmonary microvascular endothelial cells (PMVECs) is unknown but worth studying because abnormal pulmonary EC apoptosis is disadvantageous (Le et al., 2008; Sakao et al., 2005). Thus, in this study, we tested the hypothesis that physiological shear stress signals through PI3K to reduce apoptosis in human pulmonary microvascular endothelial cells.

## 2 MATERIALS AND METHODS

### 2.1 Cell culture

Primary human PMVECs (Lonza; CC‐2527) were cultured using microvascular endothelial cell complete media (Lonza; CC‐3202) on flasks (for cell expansion) or 6‐well plates (for experiments) coated with attachment factor (Gibco; S006‐100). Confluent cells at Passage 6–7 were utilized for all experiments. Donor information is listed in Table S1. Cells from both male and female donors were included in this study. For all graphs, data collected from male‐derived cells were denoted by open circles, while those from female‐derived cells were denoted by closed circles.

### 2.2 Orbital shaker model of shear stress

Primary human PMVECs were cultured in standard 6‐well culture plates using a fixed volume (depth) of 3 mL and viscosity of tissue culture media. Fully confluent cultures were used for experiments. Cells were exposed to shear stress using an orbital shaker (Labnique; MT‐201‐BX). We estimated the magnitude of shear stress (in dyn/cm^2^) exerted on human PMVECs using the extended solution to Stokes' second problem (Warboys et al., 2019):

$$\text{shear stress}=a\sqrt{\rho \mu {\left(2\mathrm{\pi f}\right)}^{3}},$$

where *a* is the gyration radius of the orbital shaker (1.1 cm), *ρ* is the density of the media (0.99 g/mL), *μ* is the viscosity of the media (0.008 poise) as determined by the Ostwald viscometer, and f is the frequency of rotation (3.83 rotations per second). In these experiments, we defined physiological shear stress as 12 dyn/cm^2^, consistent with previous studies (Tang et al., 2012; Tsubata et al., 2023). Control cells were grown under static culture conditions. In some experiments, cells were treated with the PI3K inhibitor LY294002 (Tocris 1130; 10 μM; 0.1% DMSO as vehicle) or/and the general protein kinase inhibitor staurosporine (STS; Abcam 120056; 20 nM; 0.01% DMSO as vehicle). To study the effects of shear stress on apoptosis induced by LY294002 or staurosporine, cells were first adapted to shear stress for 24 h and then treated with vehicle or drug in the presence of shear stress for another 24 h. Control cells were cultured under static conditions for 48 h with vehicle or drug present for the second 24 h.

### 2.3 Western blot analysis

Total protein was extracted from cells using lysis buffer (CST; 9803) supplemented with protease inhibitor (Roche; 11836153001) and phosphatase inhibitor (Roche; 04906837001). For each sample, protein concentration was determined using BCA protein assay (Thermo Scientific; 23225). For immunoblots, 10 μg of total protein was separated by electrophoresis using 10% SDS‐PAGE gels and transferred to PVDF membranes (Bio‐Rad; 1620177). Membranes were blocked with 5% (w/v) non‐fat milk (Bio‐Rad; 1706404) dissolved in Tris buffered saline (Quality Biological; 351‐086‐101) containing 0.2% (v/v) Tween 20 (Sigma; P7949), then incubated in primary antibodies at 4°C overnight. Horseradish peroxidase (HRP)‐conjugated anti‐rabbit (Sera care; 5220‐0336; 1:5000) or anti‐mouse (Sera care; 5220‐0341; 1:10000) secondary antibodies were utilized to detect primary antibodies. HRP reacted with ECL substrate (Bio‐Rad; 170‐5061) and chemiluminescent signals were captured by ChemiDocMP imaging system (Bio‐Rad). Membranes were probed for phosphorylated proteins first, followed by total protein and finally housekeeping protein (β‐tubulin). Densitometric analysis of protein bands was carried out using Fiji ImageJ (Schindelin et al., 2012). Primary antibody information is listed in Table S2. Phosphorylated Akt (p‐Akt) was normalized to Akt.

### 2.4 Caspase activity assessment

Caspase activity was measured using the Caspase‐Glo 3/7 Assay (Promega; G8091) following the manufacturer's instructions. Briefly, the Caspase‐Glo 3/7 reagent was reconstituted by adding Caspase‐Glo buffer to Caspase‐Glo 3/7 substrate and warmed up to room temperature before use. Twenty‐five microliter of Caspase‐Glo 3/7 reagent was incubated with 25 μL of whole‐cell lysates containing 5 μg protein in the wells of a 96‐well plate (white opaque; flat bottom). Detections were run in duplicate. Luminescence was recorded by a microplate luminometer (Veritas) every 5 min for a total of 120 min at room temperature. Values recorded at 60 min were included for subsequent analysis. Duplicated recordings from the same sample were averaged. Background (i.e., blank) was subtracted from luminescence (in random luminescence units [RLUs]).

### 2.5 Hoechst staining

Cells were stained with 2.5 μg/mL Hoechst 33342 (Invitrogen; H3570) for 15 min. Images were obtained by an investigator blinded to treatment conditions using an inverted fluorescent microscope (Olympus IX51) equipped with a 20× objective. Nine or sixteen images were captured randomly for each well. Cells with normal and condensed chromatin were automatically identified and counted by a Fiji ImageJ (Schindelin et al., 2012)‐based trained model. This model was developed by feeding a series of training images to CSB Deep in the Fiji ImageJ plug‐in (https://github.com/juglab/denoiseg/) using an Nvidia Quadro RTX 4000 GPU equipped with TensorFlow 1.15, CUDA 10.0, and cudnn 7.6.5 on Fedora 38. The training images were created by an experienced technician who manually labeled condensed and total nuclei on each image with QuPath v0.4.3. Percent apoptosis was calculated as the percentage of cells with condensed chromatin to total nuclei on each image. Counts obtained from images taken from the same well were averaged to obtain a mean value for each sample.

### 2.6 Apoptotic DNA fragmentation

Cells were trypsinized and centrifuged at 500 g for 5 min. Cell pellets were washed with PBS. Subsequent DNA isolation and electrophoresis on a 1.8% agarose gel (Invitrogen; 16500500) were carried out using the enhanced apoptotic DNA ladder detection kit (Abcam; ab66093) according to the manufacturer's protocol. Agarose gels were imaged by the ChemiDoc MP imaging system (Bio‐Rad; excitation source: UV trans illumination; emission filter: 590/110 filter).

### 2.7 Statistical analysis

Data are expressed as means ± standard deviation (SD). Technical replicates were averaged. Each “n” represents a different cell lot (i.e., biological replicate). Experiments were independently repeated in cells derived from at least three different donors. Data normality was examined with the Shapiro–Wilk test. Skew‐distributed data were log transformed. Data were analyzed by one‐way analysis of variance (ANOVA) with Dunnett's multiple comparisons test, two‐way ANOVA with Tukey's multiple comparisons test, or three‐way ANOVA with Sidak's multiple comparisons test. Statistical significance is defined by *p* < 0.05. All statistical analyses were conducted using GraphPad Prism 10 software.

## 3 RESULTS

### 3.1 Effect of shear stress on PI3K activity

**FIGURE 1:**
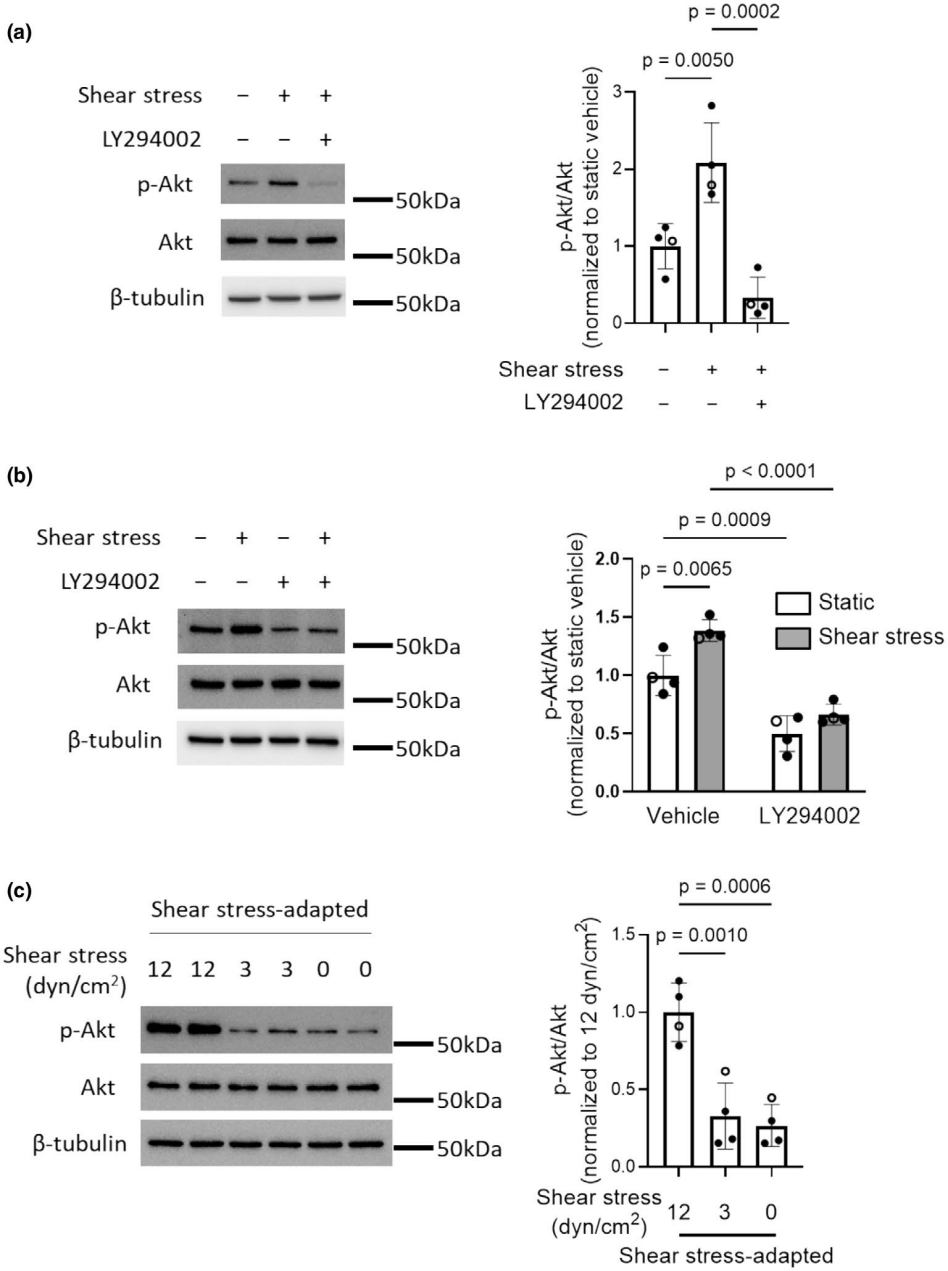
Shear stress activates PI3K/Akt in human PMVECs. Left: Representative immunoblots showing protein levels of phosphorylated Akt (p‐Akt), total Akt and β‐tubulin in whole cell lysates of human PMVECs exposed to different shear stress protocols. Right: Densitometry analysis showing corresponding ratio of p‐Akt to Akt. (a) Cells were pretreated with vehicle (0.1% DMSO) or PI3K inhibitor (LY294002; 10 μM) for 30 min and then exposed to physiological shear stress (12 dyn/cm^2^) for 1 h, with static cells as controls. (b) After adapting to 0 or 12 dyn/cm^2^ of shear stress for 24 h, human PMVECs were treated with vehicle (0.1% DMSO) or LY294002 (10 μM) while maintaining at respective static or shear stress conditions for another 24 h. (c) Human PMVECs were first adapted to shear stress (12 dyn/cm^2^) for 24 h and then exposed to 30 min of reduced (3 or 0 dyn/cm^2^) shear stress, with cells maintained at 12 dyn/cm^2^ as control. For all graphs, symbols represent different results from different donors (*n* = 4; open circles represent cells from male and closed circles represent cells from female). Bar graphs show mean ± SD values. Data were analyzed by one‐way ANOVA with Dunnett's multiple comparisons test (panel (a) interaction *p* = 0.0003 and panel (c) interaction *p* = 0.0005) or two‐way ANOVA with Tukey's multiple comparisons test (panel (b) interaction *p* = 0.1159).

To determine the effect of shear stress on PI3K activation in human PMVECs, we measured the phosphorylation of the PI3K downstream target, Akt. Compared to human PMVECs grown under static conditions, cells acutely exposed to 1 h of physiological shear stress (12 dyn/cm^2^) exhibited increased Akt phosphorylation (Figure 1a and Figure S1A), which was prevented by the specific PI3K inhibitor, LY294002. Total Akt abundance was not altered by shear stress (Figure S1B). To explore the sustainability of shear stress‐induced PI3K activation, cells were exposed to physiological shear stress for 24 h. Again, Akt phosphorylation (Figure 1b and Figure S1C) was significantly increased in cells exposed to prolonged shear stress compared to cells grown under static conditions and was attenuated by LY294002. Akt abundance was not affected by either shear stress or LY294002 in 24‐h experiments (Figure S1D). Finally, to determine whether continued exposure to shear stress was required for maintaining Akt phosphorylation in human PMVECs, cells were exposed to physiological shear stress for 24 h, after which shear stress was stopped (0 dyn/cm^2^) or reduced (3 dyn/cm^2^) for 30 min prior to measuring Akt phosphorylation. Cessation or reduction of shear stress resulted in a dramatic reduction in Akt phosphorylation (Figure 1c and Figure S1E) whereas the abundance of Akt was not altered (Figure S1F). These results show that Akt phosphorylation is rapidly and dynamically regulated by shear stress and sustained for the duration of exposure through PI3K.

### 3.2 Role of PI3K in human pulmonary microvascular endothelial cell viability

**FIGURE 2:**
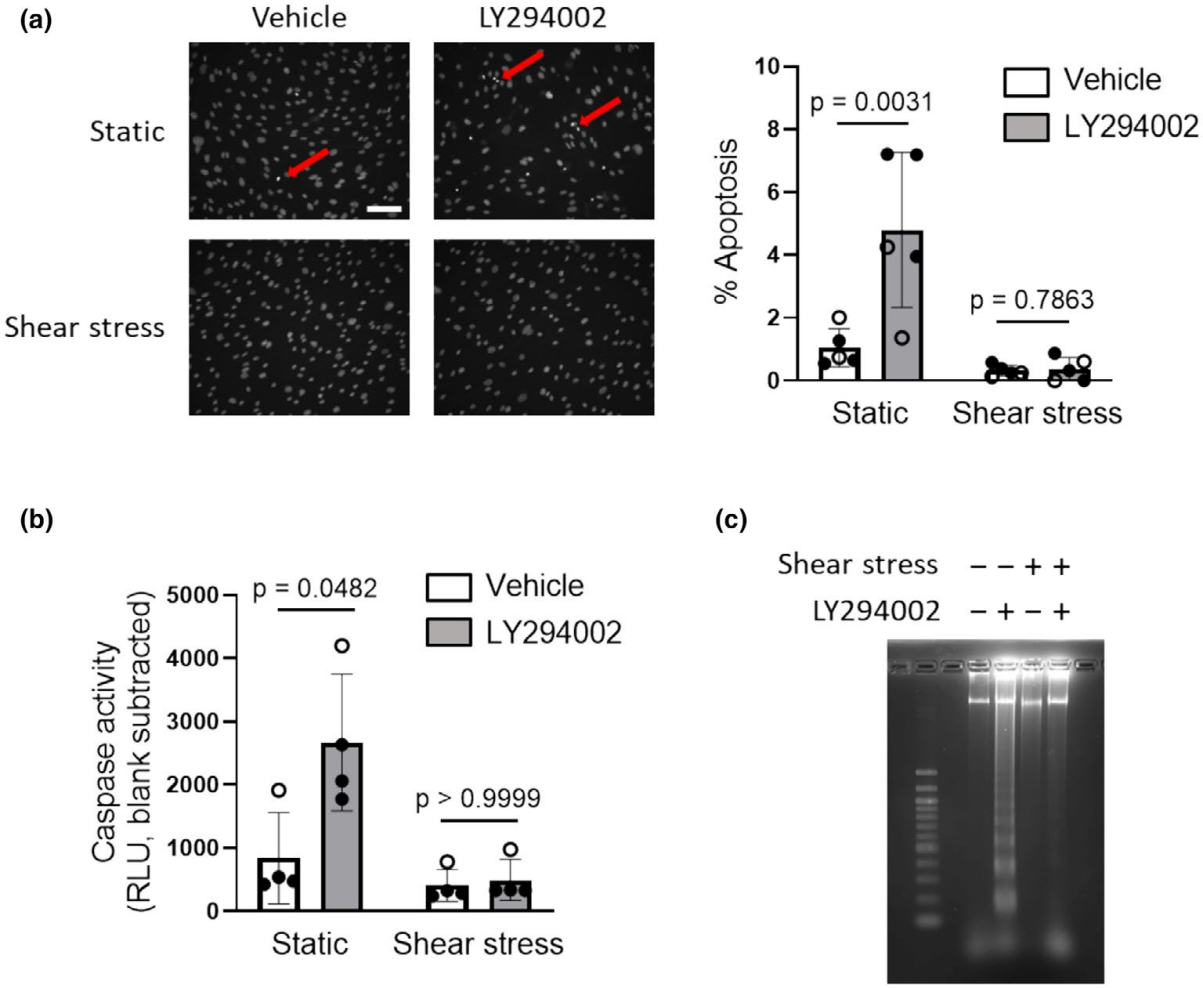
Shear stress reduces apoptosis in human PMVECs. After adapting PMVECs to 0 or 12 dyn/cm^2^ of shear stress for 24 h, cells were treated with vehicle (0.1% DMSO) or LY294002 (10 μM) while maintaining at respective static conditions or shear stress for another 24 h. Apoptosis was evaluated by (a) Hoechst staining (Left: Representative images, white scale bar is 100 μm, red arrows denote condensed chromatin; Right: Summary data); (b) caspase 3/7 activity and (c) DNA fragmentation (image representative of 3 separate experiments). Bar graphs show mean ± SD values; symbols represent results in cells from different donors (*n* = 4–5; open circles represent cells from male and closed circles represent cells from female). Data were analyzed by three‐way ANOVA (Factors are shear stress, STS and LY294002; panel (a) interaction *p* = 0.0451; panel (b) interaction *p* = 0.0393) followed by Sidak's multiple comparisons test. Shear stress groups are the same as respective panels in Figure 5.

Under static culture conditions, prolonged inhibition of PI3K activity was previously shown to induce apoptosis in HUVECs (Gerber et al., 1998). To determine the effect of blockade of PI3K on human PMVEC survival, confluent cells grown in complete growth media were exposed to the PI3K inhibitor LY294002 (10 μM) or vehicle under static conditions or shear stress for 24 h, and markers of apoptosis were measured. In cells maintained under static conditions, LY294002 significantly increased chromatin condensation (Figure 2a), caspase 3/7 activity (Figure 2b), and DNA fragmentation (Figure 2c). In contrast, LY294002‐induced apoptosis was completely suppressed in human PMVECs adapted to physiological shear stress for 24 h prior to drug exposure.

### 3.3 Staurosporine‐induced apoptosis in static and shear stress‐adapted cells

**FIGURE 3:**
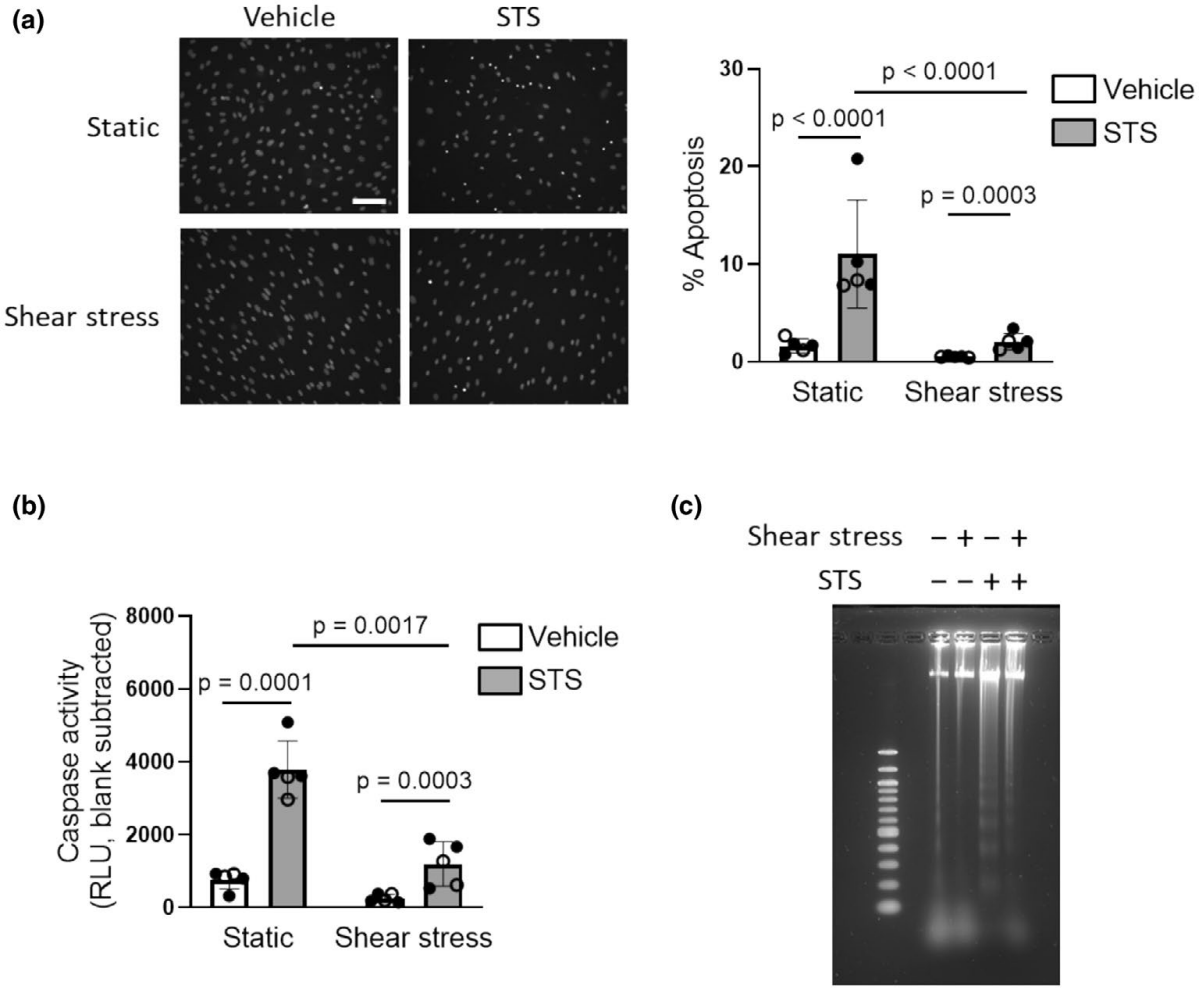
Shear stress attenuates STS‐induced apoptosis in human PMVECs. After culture under static conditions or adaptation to 12 dyn/cm^2^ for 24 h, human PMVECs were treated with either vehicle (0.01% DMSO) or staurosporine (STS; 20 nM) and maintained at their respective static or shear stress conditions for another 24 h. Apoptosis was evaluated by (a) Hoechst staining (Left: Representative images, white scale bar is 100 μm; Right: Summary data); (b) caspase 3/7 activity and (c) DNA fragmentation (image representative of 3 separate experiments). Bar graphs show mean ± SD values; symbols represent results in cells from different donors (*n* = 5; open circles represent cells from male and closed circles represent cells from female). Data were analyzed by two‐way ANOVA (panel (a) interaction *p* = 0.0830; panel (b) interaction *p* = 0.6835) followed by Tukey's multiple comparisons test.

To determine whether the anti‐apoptotic effects of physiological shear stress in human PMVECs applied to a wider range of apoptotic stimuli, cells were challenged with the broad‐spectrum protein kinase inhibitor staurosporine (Kabir et al., 2002) (STS; 20 nM; 0.01% DMSO as vehicle). Apoptosis was measured in cells cultured in complete growth media under static conditions (control) or shear stress (12 dyn/cm^2^) for 24 h, then exposed to 20 nM STS or vehicle while static conditions or shear stress were maintained for an additional 24 h. As expected, STS increased chromatin condensation (Figure 3a), caspase activation (Figure 3b) and DNA condensation (Figure 3c) in cells cultured under static conditions. However, all markers of STS‐induced apoptosis were significantly attenuated by exposure to shear stress.

### 3.4 Role of PI3K activity in shear stress‐induced apoptotic resistance to STS

**FIGURE 4:**
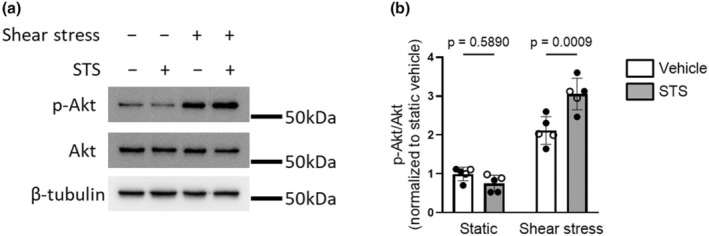
Shear stress‐induced increases in PI3K activity are not inhibited by STS. After adaptation to 0 or 12 dyn/cm^2^ for 24 h, human PMVECs were treated with either vehicle (0.01% DMSO) or staurosporine (STS; 20 nM) while maintaining at their respective shear conditions (0 or 12 dyn/cm^2^) for another 24 h. Immunoblot was performed in whole cell lysates to probe for p‐Akt, Akt and β‐tubulin. (a) Representative blot and (b) Densitometry analysis for p‐Akt to Akt ratio. Symbols represent different results from different donors (*n* = 5; open circles represent cells from male and closed circles represent cells from female). Bar graphs show mean ± SD values; Data were analyzed by two‐way ANOVA (Interaction *p* = 0.0005) followed by Tukey's multiple comparisons test.

**FIGURE 5:**
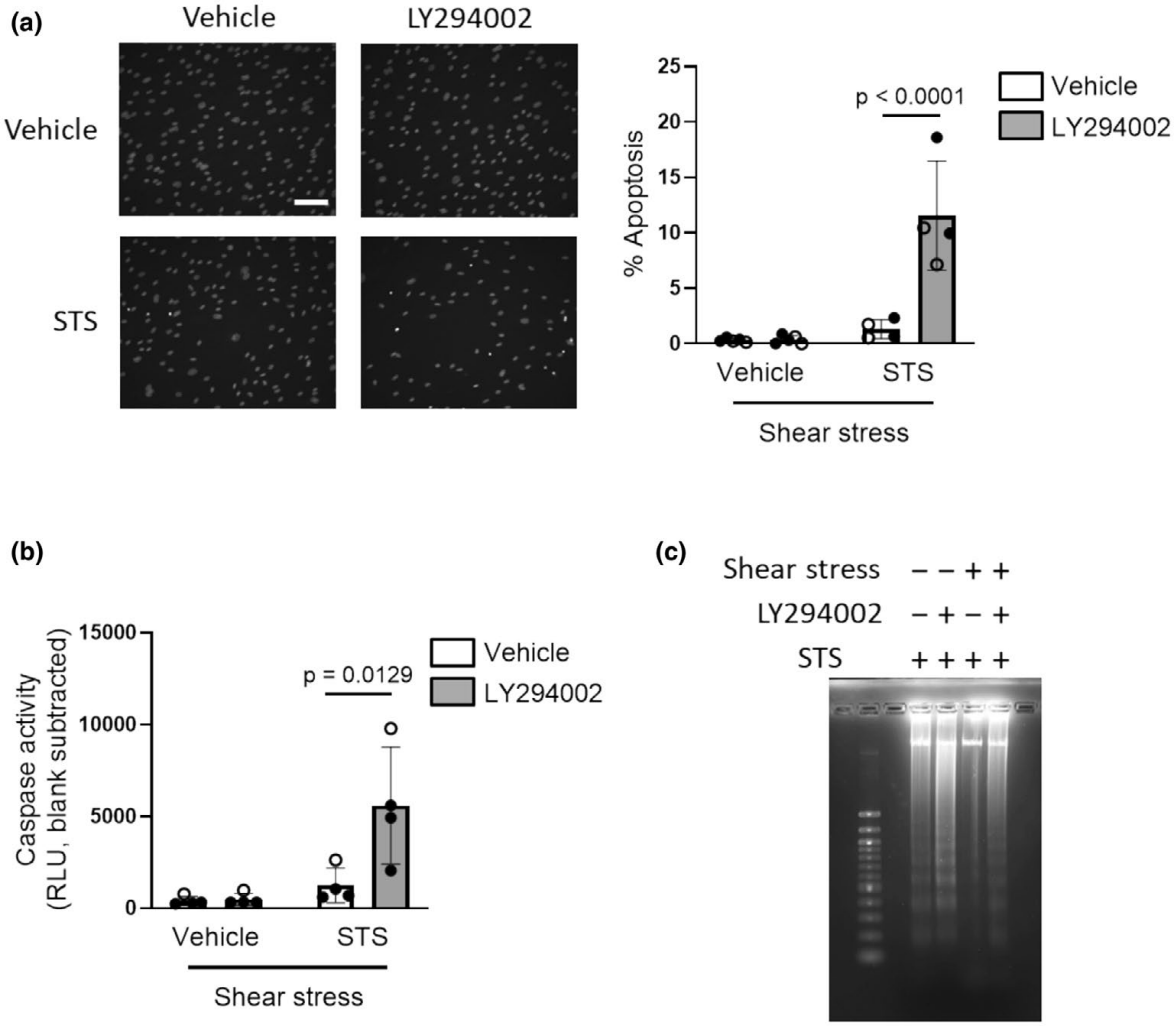
PI3K/Akt contributes to shear stress‐dependent apoptotic resistance to STS. After adaption to 0 or 12 dyn/cm^2^ of shear stress for 24 h, human PMVECs were treated with vehicle (0.1% DMSO) or LY294002 (10 μM) in combination with vehicle (0.01% DMSO) or staurosporine (STS; 20 nM) while maintaining at respective static or shear conditions for another 24 h. Apoptosis was evaluated by (a) Hoechst staining (Left: Representative images, white scale bar is 100 μm; Right: Summary data), (b) caspase 3/7 activity, and (c) DNA fragmentation (representative of three experiments). Symbols represent different results from different donors (*n* = 4–5; open circles represent cells from male and closed circles represent cells from female). Bar graphs show mean ± SD values; Data were analyzed by three‐way ANOVA (Factors are shear stress, STS and LY294002; panel (a) interaction *p* = 0.0451; panel (b) interaction *p* = 0.0393) with Sidak's multiple comparisons test. Vehicle and vehicle LY294002 groups are the same as respective panels in Figure 2.

Finally, we hypothesized that the ability of physiological shear stress to attenuate STS‐induced apoptosis was mediated by the activation of PI3K. We compared Akt phosphorylation in human PMVECs exposed to STS under static and shear‐stress conditions. Similar to results obtained in cells subjected to shear stress for shorter durations, Akt phosphorylation was increased in cells adapted to and maintained under physiological shear stress for a total of 48 h, compared to cells under static conditions (Figure 4a and Figure S2A). In a somewhat surprising result, we found Akt phosphorylation was increased slightly with STS exposure compared to vehicle‐treated cells exposed to shear stress, perhaps reflecting a compensatory anti‐apoptotic mechanism. The abundance of Akt did not change in any experimental condition (Figure S2B). In human PMVECs adapted to physiological shear stress for 24 h and then exposed to 20 nM STS and continued shear stress, inhibiting PI3K with LY294002 (10 μM) resulted in a significant increase in chromatin condensation (Figure 5a), caspase 3/7 activation (Figure 5b) and DNA fragmentation (Figure 5c) compared to cells treated with STS under shear stress alone, suggesting that PI3K mediates shear stress‐induced apoptosis resistance.

## 4 DISCUSSION

**FIGURE 6:**
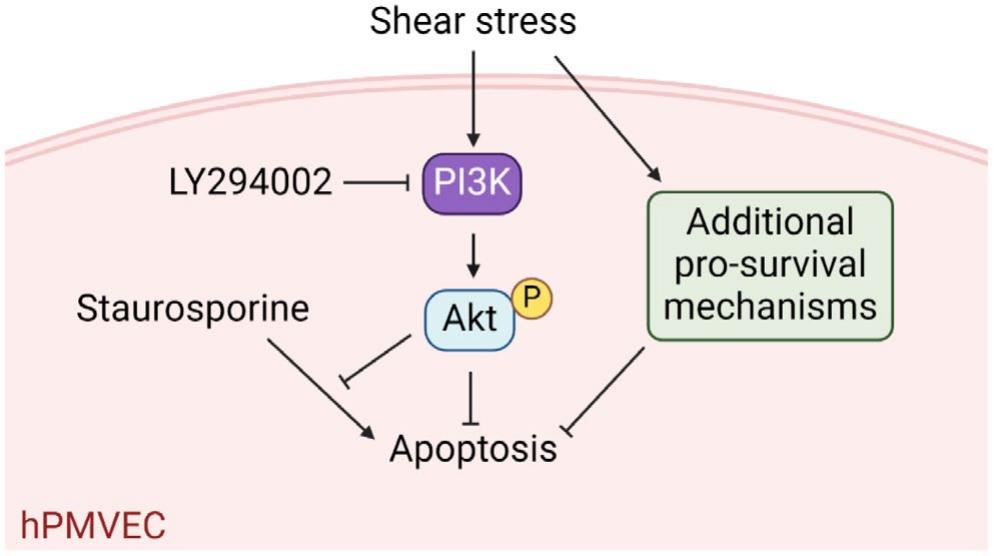
Schematic summary of major findings. In human PMVECs, physiological shear stress rapidly and sustainably activates PI3K/Akt signaling, which contributes to apoptotic resistance upon staurosporine challenge. Apoptotic resistance conveyed by shear stress is not disrupted by PI3K inhibition, suggesting the existence of additional pro‐survival pathways.

Pulmonary vascular diseases are inherently linked with alterations in blood flow and EC shear stress. Delineating mechanisms that maintain microvascular EC homeostasis under physiological shear stress is a fundamental step in understanding the potential pathogenic effects of changes in shear stress that arise in disorders like pulmonary embolism and pulmonary hypertension. In this report, we used an in vitro model and primary human cells to evaluate the effects of sustained shear stress on the susceptibility of lung microvascular endothelial cells to apoptosis. We showed in human PMVECs that PI3K activity measured by phosphorylation of the downstream target, Akt, is increased in a sustained manner by exposure to shear stress in the physiological range. Furthermore, sustained exposure to physiological shear stress protects these cells from apoptosis induced by protein kinase inhibition by LY294002 or STS and that, in the case of STS, protection is dependent on PI3K activity (Figure 6). Overall, our findings suggest an important role for physiological shear stress in the maintenance of pulmonary microvascular homeostasis and implicate PI3K as a critical mediator of shear stress‐dependent anti‐apoptotic effects.

Our findings are consistent with observations in other EC types and highly relevant to pulmonary vascular disorders like pulmonary hypertension and pulmonary embolism. Physiological shear stress has been shown to reduce apoptosis in HUVECs (Dimmeler et al., 1996, 1998; Hermann et al., 1997; Liu et al., 2015), bovine aortic endothelial cells (Haga et al., 2003; Rennier & Ji, 2015), and rat brain (Tian et al., 2013) and rat cardiac (Zhang et al., 2021) microvascular endothelial cells. In the pulmonary circulation, suppression of apoptosis under physiological conditions may have important implications when flow is disturbed. For example, dysregulation of PMVEC apoptosis is an essential element in the proposed pathogenic mechanism of PAH development, leading to the expansion of hyper‐proliferative and apoptosis‐resistant EC populations and plexiform lesions (Sakao et al., 2005). Indeed, cultured pulmonary endothelial cells obtained from subjects with idiopathic PAH showed increased rates of proliferation and reduced apoptosis when compared with cells from healthy subjects (Masri et al., 2007). Pulmonary EC apoptosis is also relevant in the pathogenesis of acute and chronic pulmonary thromboembolic disease. Zhang et al. demonstrated significantly increased apoptosis in pulmonary artery ECs isolated from rabbits after experimental pulmonary embolism (Zhang et al., 2019), which might be expected to acutely experience reduced flow, and thereby decreased shear stress. Conversely, pulmonary artery ECs isolated from subjects with chronic thromboembolic pulmonary hypertension (CTEPH) are resistant to apoptosis (Tura‐Ceide et al., 2021), similar to observations in idiopathic PAH. Furthermore, pulmonary vascular ischemia and reperfusion, obligatory with definitive therapy for CTEPH (pulmonary thromboendarterectomy surgery), are also associated with increased pulmonary EC apoptosis during both ischemia and reperfusion phases (Sage et al., 2008). Our findings confirm the critical role of sustained physiological shear stress in protecting the pulmonary circulation from EC apoptosis, and we speculate that the disruption of normal shear stress plays an integral role in disease development by promoting apoptosis and selection of apoptosis‐resistant EC populations, thereby contributing to pressure elevation.

We also identified PI3K as an important mediator of shear stress‐induced apoptosis resistance in PMVECs. Although we used a single model for apoptosis induction (protein kinase inhibition), our findings are consistent with previous observations that PI3K plays an important role in protecting PMVECs from apoptosis induced by lipopolysaccharide (Wang et al., 2022) and by renal ischemia–reperfusion injury (Li et al., 2018). PI3K converts the plasma membrane component phosphatidylinositol bisphosphate (PIP2) into phosphatidylinositol triphosphate (PIP3), which can serve as a docking site for the recruitment of pleckstrin homologous (PH) domain‐containing molecules, including phosphoinositide‐dependent kinase 1 (PDK1) and Akt. The subsequent phosphorylation of Akt by PDK1, autophosphorylation, or by other kinases favors cell survival via multiple mechanisms (Song et al., 2005). We observed sustained phosphorylation of Akt in human PMVECs under physiological shear stress, different from prior observations of transient phosphorylation in HUVECs (Dimmeler et al., 1998). This observation might support a role for Akt phosphorylation in the process of apoptosis resistance, but we did not identify specific downstream mediators of PI3K/Akt responsible for apoptosis resistance in this study. Future studies will be necessary to evaluate the specific role of Akt and other PI3K targets in shear‐mediated apoptosis suppression. Observations from previous studies support the biological plausibility of PI3K/Akt signaling in pulmonary vascular disease development. Akt phosphorylation is increased in the lungs from rats with experimental PAH (Tang et al., 2021) as well as in the lungs from mice and rats with hypoxic PH (Tang et al., 2015). Moreover, PAECs in PAH patients have increased phosphorylated Akt (Awad et al., 2024) and are apoptosis resistant (Masri et al., 2007). An unexpected finding of this study is the further Akt activation in shear‐stressed cells following STS, suggesting PI3K/Akt signaling is actively regulated by external stimuli in these cells. The lack of such a response in static cells indicates shear stress sensitizes cellular adaptations in harsh environments.

Our study has several limitations. Firstly, there are inherent limitations to the orbital shaker model that must be accounted for. The orbital shaker model does not apply uniform shear stress throughout the culture well, with centrally located cells (~10% of well surface area) experiencing a decreased magnitude of shear stress and less uniform directionality than peripherally located cells (Dardik et al., 2005; Filipovic et al., 2016). Although this variation in shear stress may lead to reduced numbers of cells exposed to the target level of shear stress, and thus an underestimation of effects, we were still able to see robust increases in kinase phosphorylation and gene expression in our experiments. We also used a blinded approach to image acquisition for apoptosis quantification that incorporated cells randomly selected throughout the well in an effort to mitigate this potential risk. Another limitation of the model is that the magnitude of shear stress applied to cells is directly related to the frequency of oscillation of the media (as outlined in the Stokes' equation, methods section). This limitation precludes the maintenance of physiological levels of shear stress at an oscillatory frequency similar to the cardiac cycle. Other experimental systems (like microfluidics or parallel‐plate configurations) could provide additional/complementary benefits, though would lose some of the strengths of the orbital shaker model. Unique strengths of the orbital shaker model particularly relevant for the current studies include the relatively high cell throughput (for molecular analyses), the ability to maintain shear stress for extended durations without culture contamination, reproducibility of the model, and the ability to use cost‐effective standard culture materials. Secondly, we chose 12 dyn/cm^2^ as our physiological level of shear stress in these experiments. Although the absolute magnitude of shear stress that human PMVECs are exposed to in vivo is unknown and likely variable, this value was selected to approximate the estimated magnitude of shear stress in the human pulmonary circulation (Dimmeler et al., 1996), is generally consistent with previous studies of physiological shear stress in PMVECs (Kostyunina et al., 2023), and represents the highest level of shear stress able to be generated in our orbital shaker model. Thirdly, we induced apoptosis in our experiments via a single mechanism, chemical inhibition of protein kinases (LY294002 and STS). It is certainly possible that the protective effects of shear stress and/or the role of PI3K are relevant only in this context, and future studies will be necessary to further delineate alternative mechanisms. Future studies that include genetic gain or loss of function for PI3K and/or Akt may provide additional insight regarding the specificity of this signaling pathway for shear stress‐induced apoptosis resistance in human PMVECs. Reassuring is the previous data demonstrating similar protective effects of PI3K/Akt on PMVECs apoptosis induced by lipopolysaccharide (Wang et al., 2022) and by renal ischemia–reperfusion injury (Li et al., 2018). Finally, our work does not explain the mechanisms linking shear stress to PI3K/Akt signaling and apoptosis resistance. Previous studies have identified various cellular hubs for mechanotransduction in endothelial cells, including a membrane complex that includes VE‐cadherin, platelet endothelial cell adhesion molecule 1 (PECAM‐1), and vascular endothelial growth factor receptor 2 (VEGFR2) (Tzima et al., 2005), and focal adhesion complexes (Bellas et al., 2002). We used our orbital shaker model in preliminary experiments with inhibitors of VEGFR2 and focal adhesion kinase; these inhibitors did not prevent shear stress‐mediated increases in PI3K activation (not shown). Additional studies aimed at clarifying these important mechanisms will require a comprehensive assessment of candidates, including integrin signaling (Urbich et al., 2002), PECAM‐1 (Fleming et al., 2005), and Src family kinases (Fleming et al., 2005).

In sum, human PMVECs derived from healthy donors are protected from apoptosis by physiological levels of continuous shear stress, and these effects are mediated by PI3K activity. These observations may have important implications for understanding the pathophysiological basis of pulmonary vascular disorders associated with alterations in blood flow.

## AUTHOR CONTRIBUTIONS

S.Y. contributed to conception, experimental design, data acquisition, data analysis and interpretation, figure and manuscript preparation; N.M.P., S.T.M., X.Y. and M.P.C. contributed to performing experiments; K.S. and M.D. contributed to data interpretation; L.A.S. and T.M.K. contributed to conception, experimental design, data analysis and interpretation, and manuscript preparation. All authors contributed to manuscript editing and reviewed and approved this manuscript. No competing interests declared.

## FUNDING INFORMATION

This work is supported by United States National Institutes of Health grants R01HL126514, R01HL159906, and T32HL007534 and by American Heart Association Postdoctoral Fellowship 24POST1194992.

## ETHICS STATEMENT

The research presented in this article did not involve human or animal subjects.

## Supporting information


Figure S1.
Figure S2.
Table S1.
Table S2.

## Data Availability

The data that support the findings of this study are available from the corresponding author (T.M.K.) upon reasonable request. All relevant data, including raw data and processed datasets, are stored securely and can be accessed for academic or research purposes following appropriate request procedures.
