# Supplementary material for: Physiological shear stress suppresses apoptosis in human pulmonary microvascular endothelial cells
Source: Physiol Rep. 2025 Mar 20;13(6):e70269. doi: 10.14814/phy2.70269 (PMC11923950; doi:10.14814/phy2.70269)
Supplement: Supplementary file 1 — Figure S1. Figure S2. Table S1. Table S2. [file PHY2-13-e70269-s001.docx]

**
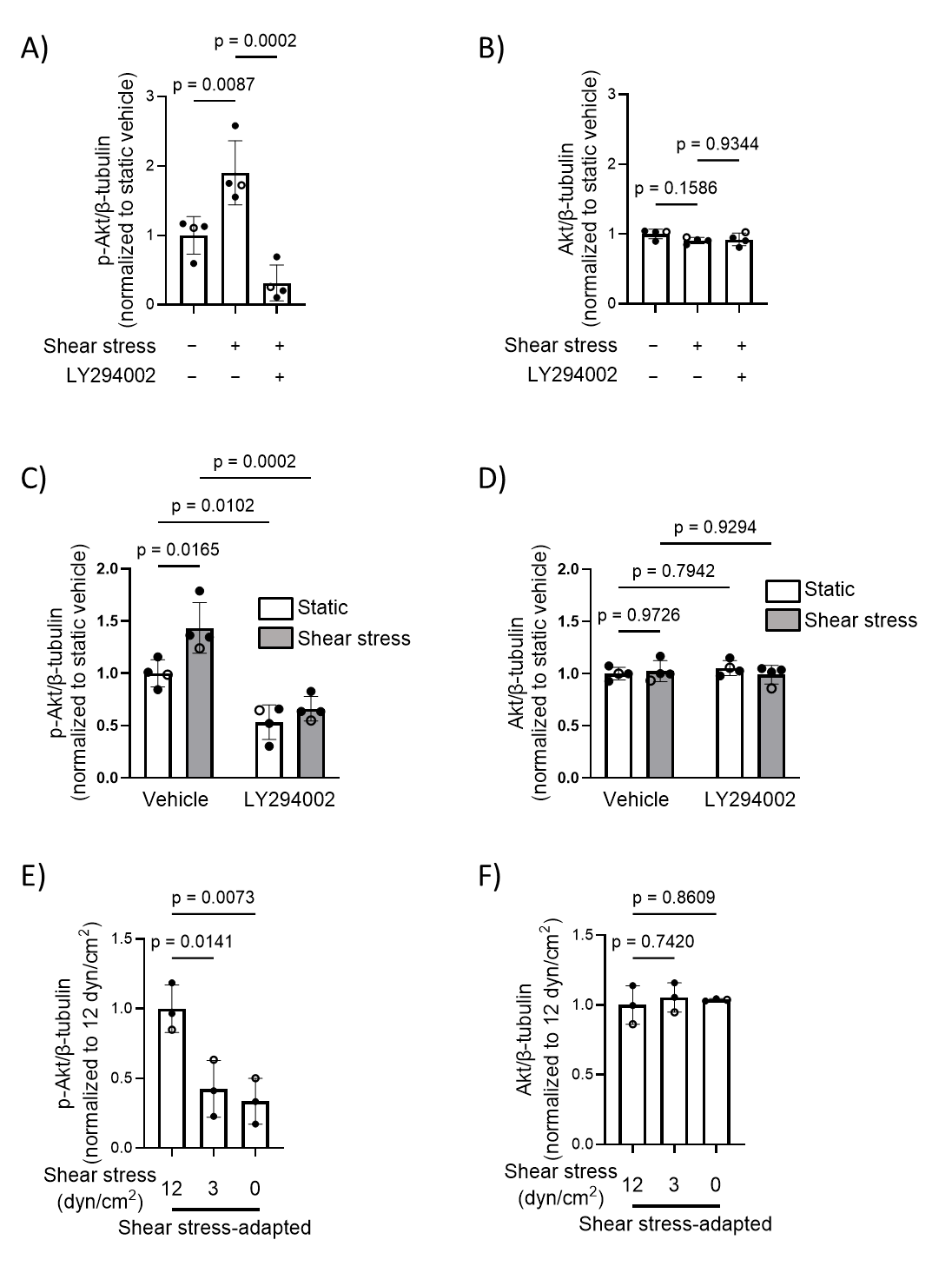
Supplemental Figure 1**. Shear stress activates PI3K/Akt in human PMVECs. Densitometry analysis of blots shown in Figure 1 showing ratio of p-Akt to β-tubulin (Panels A, C and E) or ratio of Akt to β-tubulin (Panels B, D and F) in cells subjected to different shear stress protocols as detailed below. **A)** and **B)** Cells were pretreated with vehicle (0.1% DMSO) or PI3K inhibitor (LY294002; 10 µM) for 30 min and then exposed to physiological shear stress (12 dyn/cm^2^) for 1 h, with static cells as controls. **C)** and **D)** After adapting to 0 or 12 dyn/cm^2^ of shear stress for 24h, human PMVECs were treated with vehicle (0.1% DMSO) or LY294002 (10 µM) while maintaining at respective static or shear conditions for another 24h. **E)** and **F)** Human PMVECs were first adapted to shear stress (12 dyn/cm^2^) for 24h and then exposed to 30 min of 3 or 0 dyn/cm^2^ of shear stress, with cells maintained at 12 dyn/cm^2^ as control. For all graphs, symbols represent different results from different donors (n=3-4; open circles represent cells from male and closed circles represent cells from female). Bar graphs show mean±SD values. Data were analyzed by one-way ANOVA with Dunnett’s multiple comparisons test (Panel A interaction p=0.0004; Panel B interaction p=0.1856; Panel E interaction p=0.0080; Panel F interaction p=0.2324) or two-way ANOVA with Tukey’s multiple comparisons test (Panel C interaction p=0.0984; Panel D interaction p=0.3025).


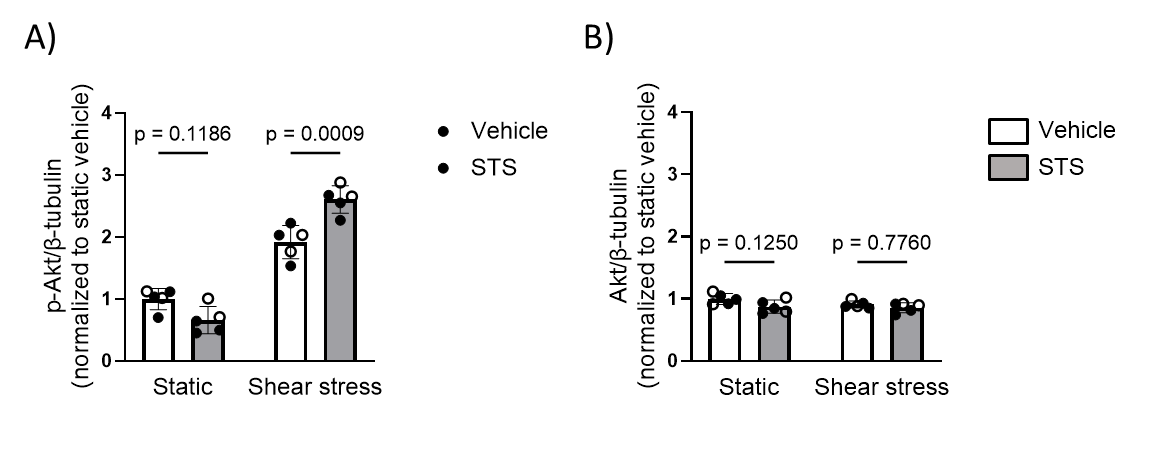


**Supplemental Figure 2**. Shear stress-induced increases in PI3K activity are further increased by STS. After adaptation to 0 or 12 dyn/cm^2^ for 24h, human PMVECs were treated with either vehicle (0.01% DMSO) or staurosporine (STS; 20 nM) while maintaining at their respective shear conditions (0 or 12 dyn/cm^2^) for another 24h. Immunoblot was performed in whole cell lysates to probe for p-Akt, Akt and β-tubulin (Figure 2). Densitometry analysis for **A)** p-Akt to β-tubulin ratio and **B)** Akt to β-tubulin ratio. Symbols represent different results from different donors (n=5; open circles represent cells from male and closed circles represent cells from female). Bar graphs show mean±SD values; Data were analyzed by two-way ANOVA (Panel A interaction p<0.0001; Panel B interaction p=0.3341) followed by Tukey’s multiple comparisons test.

**Supplemental Table 1**. Demographic information of human cell donors.

|  | Lot# | Sex | Age | Race |
| --- | --- | --- | --- | --- |
| 1 | 3F1512 | Female | 34 | Caucasian |
| 2 | 6F3497 | Male | 6 | Caucasian |
| 3 | 21TL316174 | Female | 38 | Caucasian |
| 4 | 21TL076040 | Male | 50 | Caucasian |
| 5 | 22TL024424 | Female | 43 | Caucasian |
| 6 | 22TL332326 | Female | 51 | Caucasian |
| 7 | 23TL001004 | Male | 32 | African American |

**Supplemental Table 2**. Primary antibody information.

| Primary antibody | Manufacturer, catalogue # | Source | Dilution | Diluent |
| --- | --- | --- | --- | --- |
| p-Akt ^46^ | CST, 9271 | Rabbit | 1:1000 | 5% BSA/0.2% TBST |
| Akt ^46^ | CST, 9272 | Rabbit | 1:1000 | 5% BSA/0.2% TBST |
| β-tubulin | Sigma, T7816 | Mouse | 1:10000 | 2.5% milk/0.2% TBST |
